# Exploring Antiparasitic Molecule Sources from Timber by-Product Industries—Leishmanicidal and Trypanocidal Compounds from *Clathrotropis brunnea* Amshoff

**DOI:** 10.3389/fphar.2020.584668

**Published:** 2020-12-24

**Authors:** Fernando Torres, Sara M. Robledo, Wiston Quiñones, Gustavo Escobar, Rosendo Archbold, Edwin Correa, Juan Fernando Gil, Natalia Arbeláez, Javier Murillo, Fernando Echeverri

**Affiliations:** ^1^ Group of Organic Chemistry Natural Products, Institute of Chemistry, Universidad de Antioquia, Medellin, Colombia; ^2^ PECET-Facultad de Medicina Universidad de Antioquia, Program of Study and Control of Tropical Diseases, Faculty of Medicine, Universidad de Antioquia, Medellin, Colombia

**Keywords:** antiparasite bioguided search, leishmanicidal and trypanocidal, animal model assays, flavanone and stilbene compounds, timber by-products

## Abstract

Through bioguided *in vitro* assays, the leishmanicidal and trypanocidal effects of an ethanol extract, seven fractions, and two pure substances obtained from *Clathrotropis brunnea* Amshoff sawdust were established. The effectiveness of the two metabolites was confirmed in a hamster model of cutaneous Leishmaniasis by *Leishmania braziliensis* and in Balb/c mice infected by *Trypanosoma cruzi*. *In vitro*, 3,5-dimethoxystilbene was the most active against *L. braziliensis* amastigotes, with a median lethal concentration (LC_50_) of 4.18 μg/ml (17.40 μM) and a selectivity index of 3.55, but showed moderate activity for *T. cruzi*, with a median effective concentration (EC_50_) value of 27.7 μg/ml (115.36 μM). Flavanone pinostrobin, meanwhile, showed high activity against *L. braziliensis,* with an EC_50_ of 13.61 μg/ml (50.39 μM), as well as for *T. cruzi,* with an EC_50_ of 18.2 μg/ml (67.38 μM). The animal model assay of cutaneous Leishmaniasis showed that 50% of the hamsters treated with pinostrobin were definitively cured the cutaneous ulcer, and 40% showed an improvement, with a reduction in the size of the of 84–87%. Moreover, Balb/c mice experimentally infected with *T. cruzi* and treated for 25 days with pinostrobin experienced a reduction in their parasitemia by 71%. These results demonstrate the high potential of *C. brunnea* Amshoff against cutaneous Leishmaniasis and American trypanosomiasis and indicate the pharmacological potential of waste from the wood industry, which has tons of potentially useful chemicals for the development of new medicines.

## Introduction

The WHO recently announced the 21 neglected diseases, including Leishmaniasis and trypanosomiasis, responsible for high mortality and morbidity rates, mainly in developing countries. For the management of such diseases, there are few drugs available, and those that are available have increased resistance, unpleasant side effects, and usually involve long and expensive treatments (Hotez et al., 2020). For this reason, it is necessary to look for new molecules to develop into innovative antiparasitic drugs. Paradoxically, the countries most affected by these neglected diseases have rich biodiversity, but also reduced applications and uses of these natural resources to meet their industrial, medicinal, and cosmetic needs (Cheuka et al., 2016; Luna et al., 2019).

Natural products are a significant source of new molecules and drugs. However, there are severe limitations related to the small amounts of compounds isolated from plants; this is a severe problem for molecular optimization by chemical transformation to establish structure-activity correlations and for carrying out clinical trials, as well as for commercialization. Due to this, various bioactive natural molecules remain laboratory curiosities. Proof of this is the fact that, between 1981 and 2014, only 10 drugs were approved for antiparasitic use, two of which are natural products (unmodified in structure), five were derived from a natural product, and three were made by total synthesis using a pharmacophore from a natural product (Newman and Cragg, 2016; Newman and Cragg, 2020).

Thus, our primary focus for finding antiparasitic extracts and molecules was rich sources such as the sawdust and bark of the timber industry. These plant materials represent a valuable fraction of Latin American biodiversity, including the Amazon Forest from Colombia, Brazil, Ecuador, and Perú—that are frequently used in traditional medicine. Thousands of tons of by-products also contain hundreds to thousands of kilograms of molecules potentially useful in human pharmacology, agrochemicals, cosmetics, and other types of chemical industries.

In this paper, we present the general procedures followed to detect bioactive molecules in the abundant wastes from *Clathrotropis brunnea* Amshoff against *Leishmania* and *Trypanosoma* parasites. Moreover, a bioguided assay isolated and identified two molecules that have high activity against *L. braziliensis* and *Trypanosoma cruzi* in both *in vitro* and *in vivo* studies. The flavanone pinostrobin was isolated and identified and demonstrated high efficacy in a hamster model of *L. braziliensis* in the topical application of an ointment at 6% for 28 days, as well as in a mouse model for acute/undetermined *T. cruzi* infection in oral administration at a dose of 50 mg/kg/day for 25 days. However, purification of the active substance does not appear to be a necessary process, since the extracts exhibit a level of activity akin to that of the pure substances.

## Materials and Methods

### Parasites

The *L. (V) braziliensis* strain MHOM/CO/88/UA301-EGFP (Pulido et al., 2012) and the *Trypanosoma cruzi* strain Tulahuen DTU VI transfected with the β-galactosidase gene (Buckner et al., 1996) were used. Both parasites were cultured as promastigotes and epimastigotes, respectively, at 26°C in biphasic culture medium composed of a solid phase of NNN (Novy–MacNeal–Nicholle) medium and a liquid phase composed of phosphate-buffered solution (PBS) and glucose, pH 6.9.

### Animal Model

Female and male Balb/c mice and golden hamsters (*Mesocricetus auratus*) were obtained from Charles River (United Kingdom) and then bred in-house under specific pathogen-free conditions. Animals were maintained as described previously (Robledo et al., 2012). The Animal Ethics Committee approved the infection experiments of the Universidad de Antioquia (Act 129, Aug 14, 2018).

### Collection of Plant Material

Sawdust from *C. brunnea* Amshoff were obtained in Expomaderas (Medellín, Colombia) and their identification was made by Hernán Darío Cañola at the Colegio Mayor de Antioquia, Laboratorio de Maderas; a voucher was deposited under number M1.SIU 25032020.

### Isolation and Purification

Approximately 1 kg of *C. brunnea* Amshoff (SQB-2) sawdust was extracted with ethanol (5 L) and concentrated under reduced pressure to obtain 79.5 g of dry extract. Five grams of the crude extract was then separated using Sephadex^®^ LH-20 (GE Healthcare Bio-Sciences GE Healthcare Bio-Sciences Uppsala, Sweden) with 100% methanol as eluent. Thus, seven fractions were obtained, which were submitted to antiparasitic assays.

To identify the compounds responsible of the activity, an aliquot of the fraction SQB2-S2 (450 mg) was separated by repeated preparative thin layer chromatographies in silica gel 60 F 254, 0.5 mm (Merck KGaA, Darmstadt, Germany) using petroleum ether/ethyl acetate (4.5:0.5 v/v) to obtain two pure and leishmanicidal compounds, known as SQB-2-S2-1-3 (3,5-dimethoxystilbene, 30 mg, Rf: 0.4) and SQB-2-S2-1-4 (pinostrobin, 5-hydroxy-7-methoxy-flavanone, 40 mg, Rf: 0.3).

To obtain enough pinostrobin for assays on animal models of antiparasitic activities, 13 g of crude extract was dissolved in 30 ml of 100% methanol and then filtrated. After evaporation, the extract was separated over silica gel in a glass column with petroleum ether/ethyl acetate (gradients of 48:2 v/v, 500 ml; 45:5, 500 ml; 43:7, 500 ml; 4:1, 250 ml; 3.5:1.5, 250 ml; 3:1, 200 ml; 2:1, 150 ml; 1:1, 200 ml), providing a total of 14 fractions. Then, 224.6 mg from fractions 4–7 were purified again over silica gel and petroleum ether/ethyl acetate (gradients of 48:2, 250 ml; 45:5, 250 ml; 4:1, 250 ml) to yield 84.1 mg of pinostrobin. By repeated column chromatography, approximately 600 mg of the pure compound were obtained for the leishmanicidal and trypanocidal assays.

^1^H and ^13^C NMR spectra were recorded on a Fourier 300 spectrometer (Bruker Bio-Spin GmbH, Rheinstetten, Germany), operating at 300 MHz for 1H and 75 MHz for ^13^C NMR, using CDCl_3_ (Sigma-Mo, United States) as the solvent and tetramethylsilane as an internal standard. Chemical shifts (δ) are reported in ppm, and the coupling constants (J) in Hz.

Mass spectrometry: UHR ESI–QqTOF, positive ion mode (Bruker Daltonik GmbH, Bremen Germany).

#### SQB-2-S2-1-4 (Pinostrobin, 5-Hydroxy-7-Methoxy-Flavanone)

^1^H NMR (CDCl_3_) δ (ppm): 12.08 (s, 1H) 7.48 (m, 5H), 6,12 (s, 2H), 5.46 (dd, 12.9, 3.0 Hz, 1H), 3.85 (s, 3H), 3.11 (dd, 17.1, 3.0), 2.85 (d, 17.1, 12.9).

^13^C NMR (CDCl_3_) δ (ppm), APT: 195.85 (s, C4), 168.02 (s), 164.18 (s), 162.83 (s, 8a), 138.41 (s), 128.93 (d), 126.20 (d) 103.5 (s), 95.18 (d), 94.31 (d), 79.23 (d), 55.34 (q), 43.40 (t).

HR-MS (M+H): 271.0888, calcd for C_16_H_15_O_4_ (M+H): 271.0964

#### SQB-2-S2-1-3 (3,5-Dimethoxystilbene)

^1^H NMR (CDCl_3_) δ (ppm): 7.56 (d, 7.5 Hz), 7.43 (t, 7.2 Hz), 7.33 (t, 7.2 Hz), 7.16 (d, 16.2 Hz), 7.14 (d, 16.2 Hz), 6.75 (d, 2.1 Hz), 6.47 (d, 2.1), 3.89 (s, 6H, 3.5).

^13^C NMR (CDCl_3_), δ ppm, APT: 161.02 (s), 139.40 (s), 137.17 (s), 129.25 (d), 128.76 (d), 128.71 (d), 127.81 (d), 126.65 (d), 104.62 (d), 100.02 (d), 55.43 (q).

HR-MS (M+H): 241.1145, calcd for C_16_H_15_O_4_ (M+H): 271.0964

The extracts, fractions, and molecules were dissolved in DMSO [1 mg/ml, w/v( )]. Before the *in vitro* biological assays, a stock solution of 200 μg/ml in complete RPMI-1640 medium was prepared.

### HPLC Quantitation Method Development and Calibration Curve

The crude extract solution at 1 mg/ml in methanol was used to develop the HPLC method. A C8 reverse phase column kept at 25°C (150 mm, 5 μm, 4.6 I.D. Supelcosil™, Supelco Sigma-Aldrich, St Louis MO, United States) allowed a good separation pattern of the sample. The gradient mixture of water/methanol was 90:10–50:50 (10 min), 50:50 (5 min), 50:50–20:80 (5 min), 20:80–100% methanol (5 min), 100% methanol (5 min), 100% methanol–90:10 (5 min), and 90:10 (5 min). At 1 ml/min, 10 μL of the sample was injected. DAD detection of the compound showed a maximum absorption wavelength of 215 and 290 nm; detection at 290 nm was selected to avoid noise interferences from the UV absorption of the solvents at low wavelengths (Wang et al., 2016).

Pure pinostrobin (1.0 mg/ml (3.70 mM) stock solution was prepared, and from this solution, concentrations of 10, 20, 30, and 40 μg/ml (37.02, 74.04, 111.07, and 148.09 μΜ) each were injected by triplicate, through which an equation was obtained. The calibration curve gave the equation Y = 40,008.12506 * X and *r =* 0.9953*.*

The overlapped chromatograms of the standard and the crude extract showed good resolution of the compound peak, with a retention time of 17.6 min. The crude extract was prepared at the concentrations of 2.47 and 1.57 mg/ml in methanol, and 10 μL of each solution was injected in duplicate. The active fraction SQB-2-S2 was also quantified, and 10 μL of 0.110 mg/ml was injected in duplicate.

### *In vitro* Cytotoxicity

The cytotoxicity of the compounds was evaluated according to the viability of the human promonocytic cell line U-937 (ATCC Gaithersburg, MD, United States), as described elsewhere (Upegui et al., 2020). Briefly, cells were cultured at standard conditions in RPMI-1640 medium enriched with 10% inactivated fetal bovine serum (FBS) and 1% penicillin–streptomycin solution. Cells (100,000 cells/well) in the log growth phase (3 days old) were dispensed into each well of a 96-well culture plate and then exposed to 100 μL of each of the five evaluated extract/fraction/metabolite concentrations: 200, 50, 12.5, 3.125, and 0.78 μg/ml. The plates were incubated at 37°C in a 5% CO_2_ atmosphere, and after 72 h of incubation, 10 μL/well of MTT solution (0.5 mg/ml) was added. Plates were incubated again at 37°C for 3 h. The enzymatic reaction was stopped by adding 100 μL/well of dimethyl sulfoxide. Plates were read at 570 nm in a spectrophotometer (VarioskanTM Flash Multimode Reader, Thermo Waltham, MA United States 02451). Cell viability was determined based on the quantity of formazan produced according to the intensity of the color (absorbance) registered as optical density (OD). Cells cultured in the absence of compounds were used as the control of viability (i.e., negative control), while doxorubicin was used as a control for cytotoxicity. Nonspecific absorbance was corrected by subtracting the absorbance (OD) of the blank (RPMI-1640 plus 0.2% DMSO). Assays were conducted in duplicate, with each concentration in triplicate (Upegui et al., 2020).

### *In vitro* Leishmanicidal Activity

The activity of the extracts, fractions, and pure metabolites was evaluated by flow cytometry in intracellular amastigotes of *L. braziliensis*. Briefly, U-937 human cells (300,000 cells/mL) in enriched RPMI-1640 and 0.1 μg/ml of PMA were dispensed on 24-well tissue culture plates and incubated at 37°C and 5% CO_2_. After 72 h of incubation, the cells were infected with 5-day-old growing promastigotes in a 15:1 parasite per cell ratio. The plates were incubated at 34°C and 5% CO_2_ for 3 h; then, the cells were washed twice with PBS. One milliliter of fresh enriched RPMI-1640 was added into each well, and the plates were incubated again for 24 h. Then, the culture medium was replaced by fresh enriched RPMI-1640 medium containing each extract/fraction/metabolite tested at four serial dilutions (the same used in the trypanocidal assay). The plates were incubated again at 37°C and 5% CO_2_ for 72 h. Then, the cells were removed from the bottom plate with 100 µL of a solution of trypsin/EDTA (250 mg), and the cells were centrifuged at 1,100 rpm for 10 min at 4°C, following which, the supernatant was discarded and the cells were washed with PBS. Next, the cells were suspended in 500 μL of PBS and were read at 488 nm (exciting) and 525 nm (emitting) over an argon laser of a flow cytometer (Cytomics F.C. 500MPL, Brea, CA, United States), counting 10.000 events. Infected cells were determined according to the green fluorescence events corresponding to the parasites (Newman et al., 2014). As a control, infected cells exposed to amphotericin B (AMB) were used, and infected cells not exposed to any compound or drug were used to control for 100% infection. Nonspecific fluorescence was corrected by subtracting the fluorescence of the unstained cells. Determinations were done in triplicate in at least two independent experiments.

### *In vitro* Trypanocidal Activity

To establish the effectiveness of the compounds, 25,000 U-937 human macrophages/100 μL RPMI-1640 enriched with 10% FBS and 0.1 μg/ml of phorbol 12-myristate 13-acetate (PMA) were placed into each well of the 96-wells cell culture plates. The cells were then infected with epimastigotes (24 h of growing) of *T. cruzi* in a 5:1 parasites/cell ratio. After infection, 100 μL of each extract/fraction/metabolite tested at four serial dilutions (the highest concentration being equivalent to twice the median lethal concentrations (LC_50_) were added to each well, and the plates were incubated for 72 h at 37°C and 5% CO_2_. Lastly, the effect of each compound and each concentration on the viability of the intracellular amastigotes were determined by measuring the β-gal activity by the colorimetric method after adding 100 μM chlorophenol red-β-d-galactopyranoside (CPRG; Sigma-Aldrich, St Louis, MO, United States), and nonidet P-400.1%. After 3 h of incubation at room temperature, the plates were read at 570 nm in a spectrophotometer (Varioskan, Thermo Waltham, MA 02451, United States), and absorbance was recorded as OD. The antitrypanosomal activity of benznidazole (BNZ) was used as a positive control, while RPMI-1640 medium was used as a negative control. Nonspecific absorbance was corrected by subtracting the OD of the blank sample. Determinations were done in triplicate in at least two independent experiments (Buckner et al., 1996).

### Preparation of the Pinostrobin Ointment

An aqueous solution of pinostrobin was prepared using Tween 80 5% and mineral oil 5%. In turn, the ointment was prepared based on a mixture of lanolin and petrolatum (2:1 w/w), and glycerin 1% and pinostrobin were subsequently incorporated into the base ointment until a final concentration of 6%.

### *In vivo* Antileishmanial Activity

Seven-week-old male and female golden hamsters (*M. auratus*) were infected with 25 × 10^7^ stationary growth phase promastigotes of *L. (V) braziliensis* in the dorsum as described by others (Pulido et al., 2012). After the development of the ulcer, hamsters were distributed randomly into two groups. One group (*n* = 6) was treated topically with 40 mg of an ointment formulation of 6% 5-hydroxy-7-methoxy flavanone for 28 days (defined at convenience according to the available quantity). The second group of hamsters (*n* = 3) was treated with intralesional meglumine antimoniate (MA) at 200 μg/three times per week for 4 weeks. The hamsters were monitored before treatment (TD0), at the end of the treatment (TD28), and every month post-treatment for a total of three months. The size (width and length) of the ulcer and the bodyweight were obtained at these times during the study. Since it has been shown that parasites can persist in scars (Mendonça et al., 2004; Sghaier et al., 2011), suggesting that there is no correlation between ulcer epithelialization and parasite load, the cure criterion was exclusively clinical and therefore no parasite burden was quantified.

### *In vivo* Evaluation of the Trypanocidal Effectiveness of Pinostrobin

*Mus musculus* Balb/c mice (males and females, 6–9 weeks old) were inoculated i.p with 100 μL blood containing 100 trypomastigotes of *T. cruzi* obtained from an infected mouse donor on day 24 of infection. After infection, clinical monitoring for health status and body weight was carried out weekly. Parasitemia was monitored daily by blood concentration using microhematocrit and parasite counting in the Neubauer chamber. Once the infection was confirmed (day 24 after inoculation), mice were aleatory distributed into three treatment groups (*n* = 5). One group was treated with pinostrobin at 40 mg/kg/day for 25 days (defined at convenience according to the available quantity); the second group was treated with BNZ at 100 mg/kg/day for 25 days; the third group remained without treatment (control). All mice were treated with 100 µL of the corresponding drugs or vehicle administered orally. At the end of the treatment and every month post-treatment for a total of three months, the parasitemia and the bodyweight were recorded. The state of health was monitored daily. At the end of the study, mice were euthanized for necropsy, and samples were obtained for histology analysis.

### Data Analysis

Cytotoxicity was determined according to the mortality percentages obtained for each experimental condition, i.e., extracts/fractions/molecules, AMB, BNZ, doxorubicin, and culture medium (viability control), using the following equation:

$$\text{\%Mortality}=100-\left[\frac{\text{OD}\;\text{Exposed}\;\text{cells}}{\text{OD}\;\text{Control}\;\text{cells}}\times 100\right]$$

The trypanocidal activity was calculated according to a decrease in the OD using the following equation:

$$\%\text{ reduction}\;\text{of}\;\text{parasites}=100-\left[\frac{\left(\text{OD}\;\text{infected}\;\text{treated}\;\text{cells}\right)}{\left(\text{OD}\;\text{infected}\;\text{non}-\text{treated}\;\text{cells}\right)}\times 100\right]$$

The leishmanicidal activity was calculated by a reduction in the AFU (Active Fluorescent Units) using the following equation:

$$\%\text{reduction}\;\text{of}\;\text{parasites}=100-\left[\frac{\left(\text{AFU}\;\text{infected}\;\text{treated}\;\text{cells}\right)}{\left(\text{AFU}\;\text{infected}\;\text{non}-\text{treated}\;\text{cells}\right)}\times 100\right]$$

These percentages of cell mortality were used to calculate the LC_50_, while the percentages of the reduction of parasites data were used to calculate the EC_50_ by the linear regression model Probit using Graphpad Prism software.

The cytotoxicity was defined according to the LC_50_ or HC_50_ values, using the follow scale: Toxic/hemolytic, LC_50_/HC_50_ < 100 μg/ml (extracts or fractions) or μM (pure compounds); moderately toxic, LC_50_/HC_50_ > 100 μg/ml or μM and <200 μM or μg/mL; potentially nontoxic, LC_50_/HC_50_ > 200 μg/ml or μM.

The *in vitro* leishmanicidal and trypanocidal activity were graded as high, moderate, or low according to the EC_50_ values, as follows: High activity when EC_50_ < 25 μg/ml or μM; moderate activity when EC_50_ = 25–50 μg/ml or μM; low activity when EC_50_ > 50 μg/ml or μM. The selectivity index (SI) was calculated by dividing the cytotoxic activity and the antiparasitic activity using the following formula: SI = LC_50_/EC_50_.

The *in vivo* leishmanicidal effectiveness was determined based on the reduction in the size of the ulcer in response to the treatment. For this, lesion sizes before and after treatment for every individual were compared. At the end of the study, the clinical outcome was recorded as either *cure* (epithelial healing and emergence of fur), *improvement* (reduction in the lesion size by at least 20%), *relapse* (reactivation of the lesion after initial improvement or cure), or *failure* (an increase in the lesion size). Similarly, the trypanocidal effectiveness in the mouse model was determined by the reduction in parasitemia after treatment. The clinical outcome was graded *cure* when the parasitemia was reduced by 100%, *improvement* if it was reduced by any percentage lower than 100%, and *failure* when parasitemia increased.

### Ethical Issues

The experimental procedures followed the practices, procedures and requirements for the safe handling and use of biohazardous materials for research activities specified in the Biosafety Manual of the research groups responsible for the present research.

The procedures involving animals were in compliance with the Colombian Law of Use and care of Animals in experimentation (Ley 84 del 27 Diciembre de 1989) and had the approval of the Ethical Committee for the Experimentation in animals of the Universidad de Antioquia (Act No. 123 of March 22, 2019).

## Results

### Isolation and Structure Assignments

One crude extract (SQB-2), seven fractions (SQB-2-S0 to SQB-2-S6), and two metabolites (SQB-2-S2-1-3 and SQB-2-S2-1-4) were obtained. The raw ethanolic extract of *C. brunnea* Amshoff showed high activity against the amastigotes of *L. braziliensis* (EC_50_ = 15.4 μg/ml) and *T. cruzi* (EC_50_ = 25.0 μg/ml)- but also exhibited remarkable cytotoxicity for U-937 cells that serve as hosts of both parasites, with an LC_50_ of 10.3 g/ml; therefore, the SI values were low, i.e., 0.67 and 0.4 (Table 1). The de-replication of the extract by chromatographic separation with Sephadex LH-20 resulted in seven fractions. The SQB-2-S1 and SQB-2-S3 fractions only showed good activity against *T. cruzi,* with EC_50_ values of 22.8 and 17.0 μg/ml, respectively but low activity against *L. braziliensis*, with EC_50_ values > 50 and 145.9 μg/ml, respectively. In contrast, the SQB-2-S2 fraction showed high activity against both parasites, with EC_50_ values of 13.3 and 15.7 μg/ml, respectively, and its cytotoxicity was almost three times lower (LC_50_ = 37.16 μg/ml). Given the high activity shown by the SQB-2-S2 fraction for both parasites, it was decided to continue the active metabolites isolation phase with this fraction.

**TABLE 1:** *In vitro* activity of *Clathrotropis brunnea* Amshoff on *L. braziliensis* and *T. cruzi* amastigotes and its cytotoxicity toward U-937 macrophages.

Sample	Code	U-937 cells LC_50_ (μg/ml)^a^	*L. braziliensis*		*T. cruzi*		
			EC_50_ (μg/ml)^b^	SI^c^	EC_50_ (μg/ml)^b^	SI^c^	
Crude extract	SQB-2	10.3 ± 0.7	**15.4 ± 2.7** ^d^	0.7	**25.0 ± 5.2**	0.4	
Fractions	SQB-2-S0	232.6 ± 70.3	88.4 ± 25.0	2.6	36.8 ± 0.7	6.3	
	SQB-2-S1	128.2 ± 16.4	>50	<2.6	**22.8 ± 1.0**	5.6	
	SQB-2-S2	37.2 ± 13.8	**13.3 ± 1.6**	2.8	**15.7 ± 0.9**	2.4	
	SQB-2-S3	284.2 ± 137.3	145.9 ± 48.2	2.0	**17.0 ± 1.9**	16.7	
	SQB-2-S4	62.2 ± 1.5	>50	<0.04	35.6 ± 4.3	1.8	
	SQB-2-S5	153.4 ± 44.7	>50	<3.1	36.2 ± 1.3	4.2	
	SQB-2-S6	71.2 ± 5.0	>50	<1.4	27.6 ± 2.2	2.6	
3,5-diOMe stilbene	SQB-2-S2-1-3	14.8 ± 0.7 (61.6 μM)	**4.2 ± 0.21 (17.0** μM)	3.6	27.7 ± 3.8 (115.4 μM)	0.6	
Pinostrobin	SQB-2-S2-1-4	35.5 ± 9.0 (131.4 μM)	**13.6 ± 0.28 (50.4** μM)	2.6	**18.2 ± 0.2 (67.4)**	2.0	
Amb^e^		36.6 ± 8.0	0.3 ± 0.1	122.0	NA^f^	NA	
Bnz^g^		>200.0	NA	NA	15.8 ± 2.6	>12.7	
Dox^h^		1.0 ± 0.2	NA	NA	NA	NA	

Data represent the mean value ± standard deviation. Bold values are active compounds.

a LC_50_: Lethal concentration on U-937 cells.

b EC_50_: Effective concentration on intracellular amastigotes of L. braziliensis or T. cruzi.

c SI: Selectivity index (LC_50_/EC_50_).

d High activity: EC_50_ < 25 μg/ml; moderate activity: EC_50_ > 25 < 50 μg/ml; low activity: EC_50_ > 50 μg/ml.

e Amphotericin B: Leishmanicidal drug control.

f NA: Not applicable.

g Benznidazole: Trypanocidal drug control.

h Doxorubicin: Toxicity drug control.

Through repeated chromatographic processes, two substances were obtained; the first, corresponding to the 3,5-dimethoxystilbene, was the most active against the amastigotes of *L. braziliensis,* with an LC_50_ of 4.2 μg/ml (17.4 μΜ) and an SI of 3.6, but showed moderate activity for *T. cruzi*, with an EC_50_ value of 27.7 μg/ml (115.36 μΜ). The second molecule was the flavanone pinostrobin, with an EC_50_ of 13.6 μg/ml (50.4 μΜ) for *L. braziliensis* and 18.2 μg/ml (67.4 μΜ) for *T. cruzi*. On the other hand, the activity shown by pinostrobin in *L. braziliensis* was similar to the extract from which it was previously obtained, and for *T. cruzi,* it increased by 27% since the EC_50_ values decreased from 25.0 to 18.2 μg/ml (67.4 μΜ).

**FIGURE 1:**
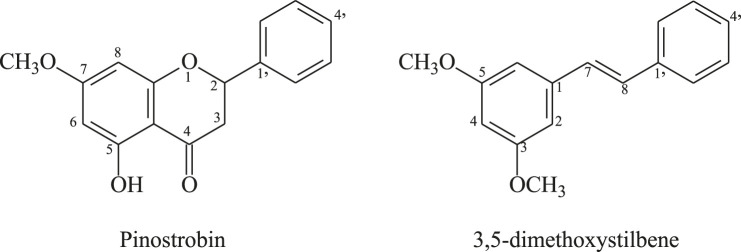
Structure of the antiparasitic compounds isolated from *C. brunnea* Amshoff sawdust.

The compound structures were assigned based on HRMS and 1D and 2D NMR (Supplementary Figure S1, Figure 1). The compound 5-hydroxy-7-methoxy flavanone (pinostrobin) has previously been reported in the literature (Cifuentes et al., 2020) and 3,5-dimethoxystilbene, was isolated from several plants, including *Alpinia katsumadai* (Ngo and Brown, 1998).

The pinostrobin content in the extracts was also analyzed by HPLC, demonstrating a presence of 10.07% in SQB-2-S2 and 0.68% in the raw extract, thus indicating that separation by Sephadex is an appropriate process for its concentration, but with reduced cytotoxicity.

The flavanone pinostrobin was selected for assays in the animal model of *T. cruzi* infection and cutaneous Leishmaniasis, as it can be found in higher concentrations in *C. brunnea* Amshoff extract, and the separation seems to be faster and easier. Semi-preparative HPLC was used to obtain the necessary amount of pure substance for the animal model assays.

### Antileishmanial Response of Pinostrobin in the Hamster Model of Cutaneous Leishmaniasis by *L. braziliensis*

**FIGURE 2:**
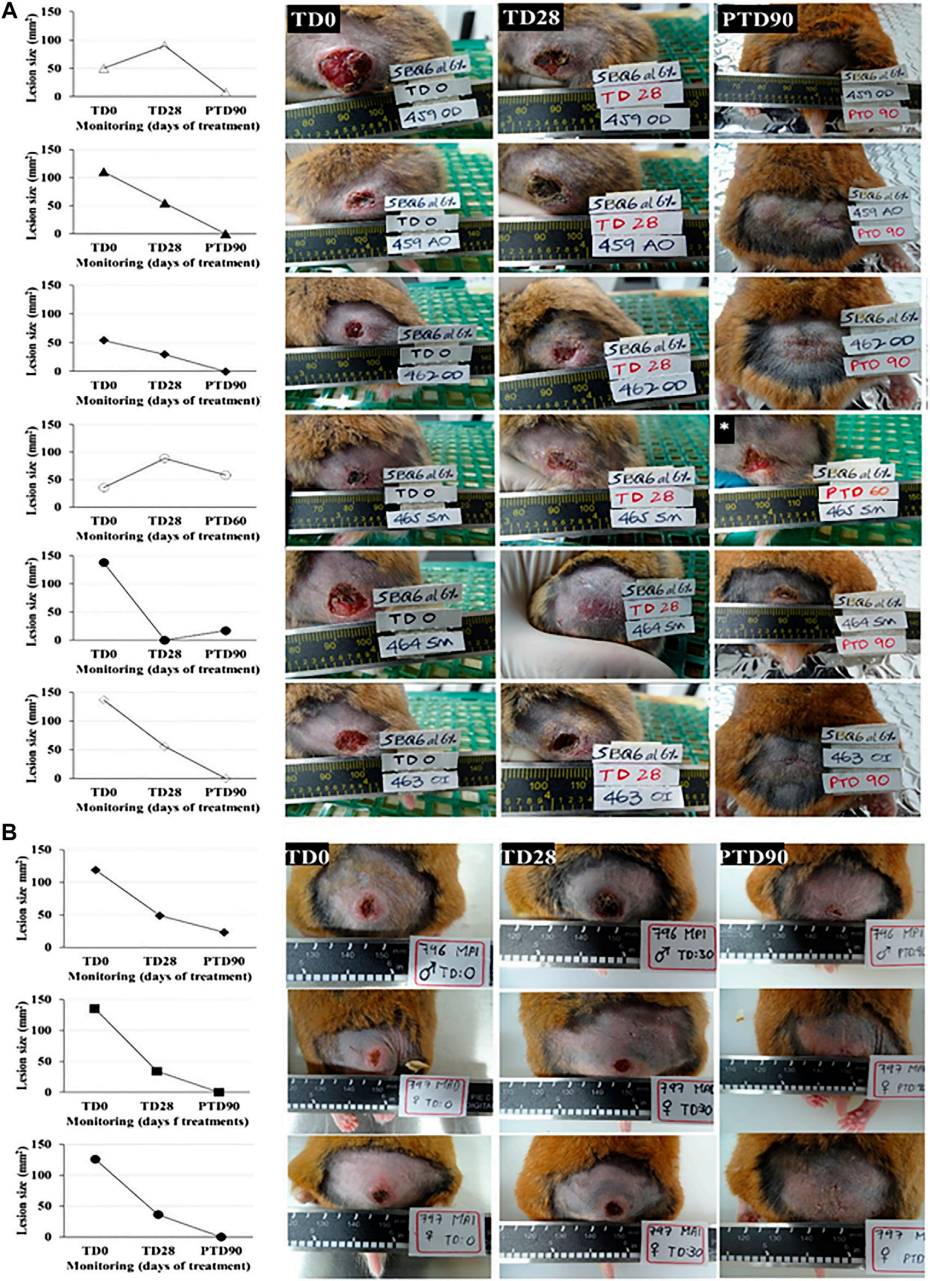
*In vivo* antileishmanial efficacy of 6% pinostrobin against *L. braziliensis* in a chronic infection model of cutaneous leishmaniasis in golden hamsters. The figure shows the progression of the disease in hamsters treated with 6% pinostrobin ointment **(A)** or intralesional meglumine antimoniate (MA) **(B)**. Disease progression was monitored before treatment (at day 0; TD0), at the end of the treatment (at day 28; TD28), and 90 days after treatment ended (PTD90). The ointment (40 mg) containing 6% pinostrobin was administered once daily for 28 consecutive days. MA was administered via an intralesional injection three times per week for four weeks. *y*-axis: Lesion size (mm^2^) for each hamster in pinostrobin **(A)** or MA **(B)**; *x*-axis: Monitoring (days).

Forty milligrams of an ointment with 6% pinostrobin was topically administered once daily for 28 consecutive days. Five of the six hamsters (83.3%) responded favorably to the treatment with pinostrobin ointment (6%), three of these hamsters (50%) were completely cured, and in the other two hamsters, their lesion decreased by 87.7% and 84.1%. The remaining hamster, although cured by the end of the treatment, had a reactivation one month later (Figure 2).

**FIGURE 3:**
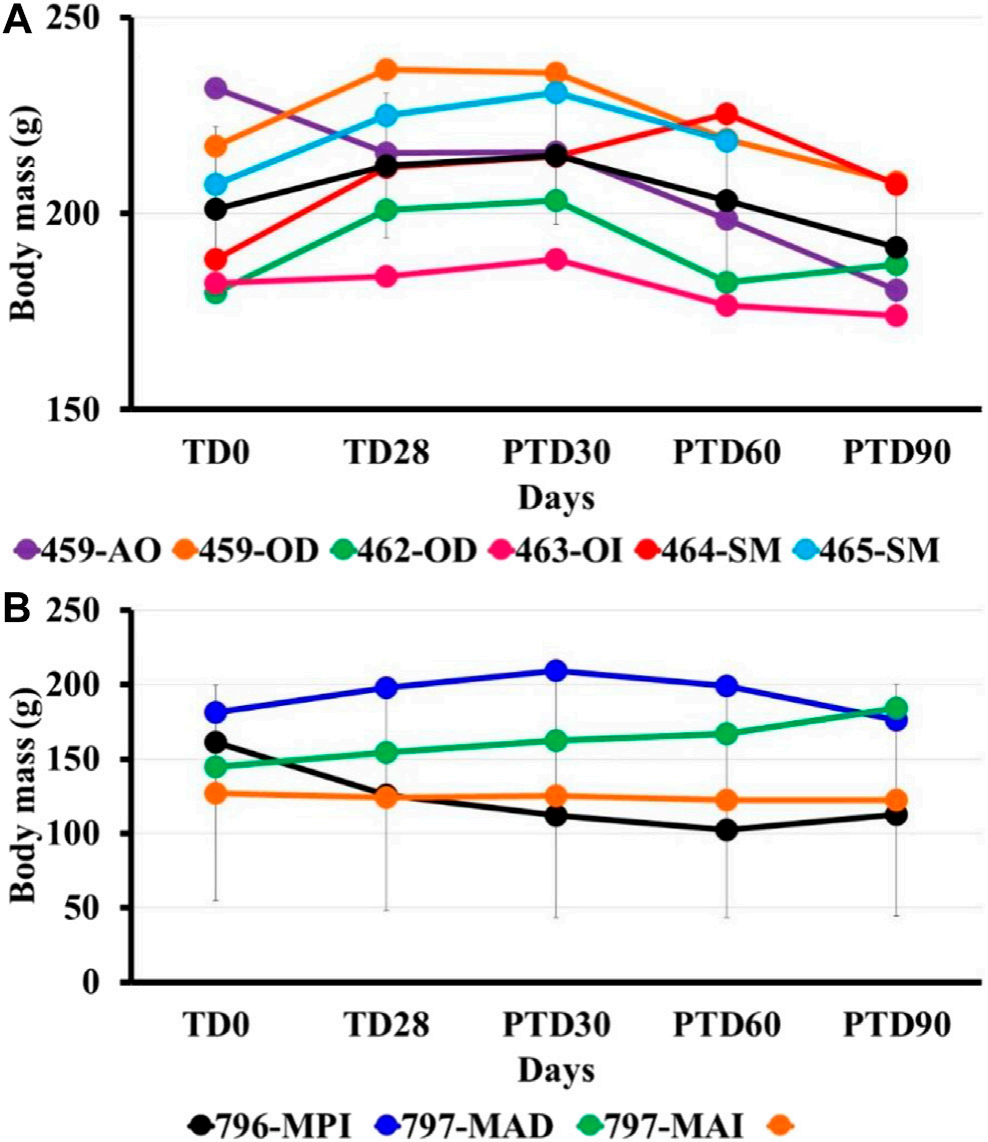
Bodyweight post-treatment using pinostrobin ointment (6%) in hamsters with cutaneous Leishmaniasis by *L. braziliensis*. The figure shows the mass bodyweight in hamsters treated with 6% pinostrobin ointment **(A)** or intralesional meglumine antimoniate (MA) **(B)**. The body weight was monitored before treatment (at day 0; TD0), at the end of the treatment (at day 28; TD28), and days 30, 60 and 90 after treatment ended (PTD30, PTD60, and PTD90, respectively). The 6% pinostrobin ointment (40 mg) was administered once daily for 28 consecutive days, and MA was administered three times per week for four weeks. *Y*-axis: Weight (g) for each hamster in the pinostrobin **(A)** or MA **(B)** groups; *x*-axis: Monitoring (days). The black line corresponds to the average body weight in the corresponding group.

No evident signs of toxicity were observed in the animals at the end of the study. The mean weight in the treated group decreased slightly, with a loss of 4.3 g on average at the end of the study (Figure 3).

### Trypanocidal Effectiveness of Pinostrobin in Balb/C Mice Infected With *T. cruzi*

**FIGURE 4:**
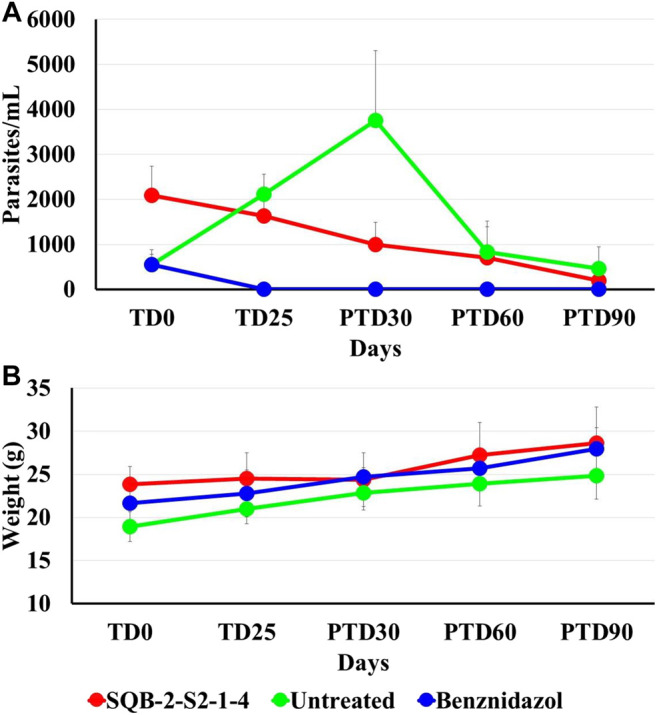
Effect of pinostrobin in parasitemia and body weight of Balb/c mice infected with *T. cruzi.* The figure shows the average number of parasites per milliliter of blood, using the microhematocrit concentration method **(A)** and the average of the animal weight in grams, of all the test groups at the beginning of the treatment (TD0), at the end of treatment (TD25), and during at days 30, 60 and 90 post-treatment follow-up (PTD 30, PTD 60, and PTD 90) **(B)**.

The parasitological evaluation showed a reduction in the levels of parasitemia in those animals treated with pinostrobin (SQB-2-S2-1-4) at the end of treatment and during the subsequent follow-up. The number of blood parasites decreased by 21.6%, and after 30, 60, and 90 days of follow-up, it was reduced by 52.0%, 66.4%, and 90.4%, respectively, in comparison to parasitemia before the treatment (Figure 4A).

In the group treated with BNZ, animals were complete cured by the end of the therapy and remained so during the post-treatment follow-up. In contrast, in the nontreated mice, there was an increase in parasitemia. Nevertheless, there was a significant reduction in parasitemia in terms of the number of parasites in blood on day 30 post-treatment, which is a normal process considering that at that time of infection (approximately 120 days after inoculation), parasites enter into a latency period. The reduction in parasitemia in the animal group treated with pinostrobin in comparison to the parasitemia showed before treatment was 21.6% at the end of treatment and 52.0%, 66.4% and 90.4 at 30, 60, and 90 days’ post-treatment, respectively.

Clinical monitoring was performed daily alongside the administration of the substances. Body weight was recorded to monitor losses and to adjust the compound volumes based on changes in weights. None of the studied mice showed alterations with the administration of the treatments, nor did they show severe clinical signs, which allowed completing the therapeutic scheme for all groups. The evolution of body weight gain remained stable throughout the trial, increasing as expected (Figure 4B). At the end of the trial, corresponding to post-treatment day 90 (PTD90), all mice were euthanized, and each mouse was necropsied. Congestion was observed in the spleen, which is a consequence of the infection, but no macroscopic abnormalities were observed in any organ that were indicative of toxicity.

## Discussion and Conclusion

The search for new antiparasitic drugs, especially leishmanicidal and trypanocidal compounds, is necessary for many countries worldwide due to the low number of drugs available, the microbial resistance and side effects, and various other factors. However, the pharmaceutical industry does not invest in such drug discovery processes because of the few gains that can be made. For this reason, countries with high parasitosis rates should be implementing efforts to find new molecules and to carry out their development into medicines. Furthermore, the WHO recommends the examination of topical formulations for skin leishmaniasis (World Health Organisation, 2010), as well as for new medicines for the oral treatment of Chagas disease (Kawaguchi et al., 2018).

Natural products are a source of medicines and innovative structures. However, restrictions in the discovery and development of new drugs are low concentrations found in plants and the lack of abundant and renewable raw materials for their production. The discovery of new bioactive molecules can take, as a basis, the existence of rich plant biodiversity in many countries affected by parasitosis, specially Leishmaniasis, and trypanosomiasis, and specifically in Asia, Africa, and Latin America. Timber industry residues, including sawdust, bark, branches, and foliage, are a good alternative, as they are produced in tons and belong to plant families rich in secondary metabolites. For this reason, in this work, we analyzed the leishmanicidal and trypanocidal effects of wood tree sawdust *C. brunnea* Amshoff, which could eventually allow access to large amounts of bioactive molecules, and thus are potential sources for the development of new medicines.

Here and through bioguided assays, we explored 15 by-products of trees against several parasites (data not shown), including *L. braziliensis* and *T. cruzi* (the causal agent of Chagas disease). In this way, the extract of *C. brunnea* Amshoff sawdust was analyzed firstly as leishmanicidal, and after *in vitro* assays and purification, an active chromatographic fraction against *L. braziliensis* was detected, with an EC_50_ of 13.32 μg/ml and an LC_50_ of 37.16 μg/ml (Figure 2). Again, given the high activity shown by the SQB-2-S2 fraction for both parasites, we decided to continue the active metabolites isolation phase with this fraction.

The main metabolite of the active chromatography fraction SQB-2-S2 was isolated and identified by spectroscopy methods like the flavanone pinostrobin; the leishmanicidal activity of the pure compound was similar to the extract, thus probably indicating the presence of other compounds with similar biological activity. The application of this compound as a 6% ointment in *L. braziliensis*-infected hamsters, once daily for 28 days, allowed to totally cure the disease in three out of five hamsters and improvement in the two remaining hamsters observed, with a reduction in the size of the ulcer by 84.1% and 87.7% (Figure 3).

On the other hand, the raw extract of *C. brunnea* Amshoff was also effective *in vitro* against the amastigotes of *T. cruzi*, with an EC_50_ of 25.0 ± 5.2 μg/ml, and the active fraction SQB-2-S2 also showed an EC_50_ of 15.7 ± 0.9 μg/ml. Oral treatment applying 40 mg/kg/day of pinostrobin for 25 days sequentially reduced parasitemia from 52.0% in the first month to 90.4% at three months (Figure 4). A substantial reduction in parasitemia of 14.9%, 30.0%, and 71.42% was achieved 30, 60, and 90 days post-treatment, respectively.

However, obtaining a pure product is not of pharmaceutical interest since industrial scale purification processes are long and costly. It is also important to note that the chromatographic fraction SQB-2-S2 showed similar activity to that obtained with pure pinostrobin for *L. braziliensis* and *T. cruzi*, 13.3 μg/ml vs 15.7 μg/ml for fraction and, 13.6 μg/ml vs. 18.2 μg/ml, respectively. For this reason, this study also focused on the effects of extracts, which are easier to produce, and which may eventually have a better activity or minor cytotoxicity. The raw extract of *C. brunnea* Amshoff had *in vitro* leishmanicidal activity of 15.4 ± 2.7 μg/ml and *in vitro* trypanocidal activity of 25.0 ± 5.2 μg/ml. In the next stage of purification using a Sephadex LH-20 chromatography column, an EC_50_ of 13.3 ± 1.6 μg/ml was found for *L. braziliensis* and of 15.7 ± 0.9 μg/ml for *T. cruzi* in the SQB-2-S2 fraction. In terms of composition, the analysis of the concentration by HPLC of the *in vivo* active flavanone pinostrobin was increased from 0.57 ± 0.007% to 0.68 ± 0.014% in the raw extract to 10.07 ± 0.23% in the SQB-2-S2 fraction.

Pinostrobin was isolated from dichloromethane extracts from *Eugenia matossi* (Myrtaceae) and showed *in vitro* activity against *L. amazonensis* and *L. braziliensis*, with an EC_50_ of 22.7 and 11.3 μg/ml, respectively in the latter parasite, similar to the value found by us (13.6 × 0.28 μg/ml), thus reconfirming the leishmanicidal activity observed (Vechi et al., 2020). It was also reported to be active in *Lychnophora markgravii* (Vernonieae, Asteraceae) against *L. amazonensis* amastigotes as well as the analog flavanone tectochrysin. (Salvador et al., 2009). Moreover, the pinostrobin isolated from the methanolic extract of the leaves of *Lychnophora staavioides* Mart. showed low activity against *T. cruzi* trypomastigotes, with a percentage of inhibition of 14.7% at a concentration of 500 μg/ml (Takeara et al., 2003).

The other compound isolated from *C. brunnea* Amshoff was 3,5-dimethoxystilbene and although has not been reported like a leishmanicide, several analogs such as resveratrol (3,5,4′-trihydroxy stilbene) and pterostilbene (3,5-dimethoxy-4′-hydroxy stilbene) have shown some *in vitro* activity. The last molecule was reported against the promastigotes and amastigotes of *L. amazonensis,* showing an EC_50_ of 17.7 and 33.2 µM, respectively. However, its mechanism of action did not affect the Sub-G0 phase or cells in the G0–G1 phase, nor did it influence the mitochondrial membrane potential (ΔΨ), which was observed for piceatannol and the structural analog (Passos et al., 2015).

Therefore, a standardized composition of *C. brunnea* Amshoff extracts containing pinostrobin may be equally effective but can be obtained more quickly and cheaply than via several exhaustive purifications of pure molecules. All of this would allow for economically and logistically feasible industrial scaling in countries that have this resource and need the application of drugs in their populations affected by *Leishmania* or *Trypanosoma* parasites. Nevertheless, it is also possible to obtain pinostrobin by chemical synthesis as a mixture of optical isomers, but the activity of each of them is not yet known.

Moreover, the antiparasitic activity of pinostrobin and 3,5-dimetoxystilbene can also be optimized in two mainly ways, chemical and therapeutic. In the first case by structural transformations to identify the pharmacophore and to modify functional groups or the type of rings. In the second case, changing the therapeutic scheme, so increasing the administered concentration and frequency of application and even modifying the formulation to achieve high penetrability in the skin, as in leishmaniasis. This is especially important against *T. cruzi* due to the small number of medicines available for its control.

In short, in this work and through bioguided assays with the sawdust of *C. brunnea* Amshoff*,* the flavanone pinostrobin was isolated and identified, demonstrating high efficacy in an animal model of cutaneous Leishmaniasis by *L. braziliensis,* as well as in an animal model of *T. cruzi* infection. However, purification of the active substance does not appear to be necessary since extracts exhibit the same level of activity as the pure substances. Moreover, these extracts contain the stilbenoid pterostilbene, which is also active against these parasites. Studies are currently being carried out to scale the production of these compounds in a faster and more efficient to obtain material for continuing the development of a medicine for the treatment of both cutaneous Leishmaniasis and Chagas Disease.

## Author’s Note

Luis Villarroel *in memoriam*, Professor of Natural Products, University of Santiago de Chile.

## Data Availability Statement

The raw data supporting the conclusions of this article will be made available by the authors, without undue reservation, to any qualified researcher.

## Ethics Statement

The animal study was reviewed and approved by Animal Ethics Committee of Universidad de Antioquia (Act 129, August 14, 2018).

## Author Contributions

FT, supervision, and reports evaluation; FT, FE, EC, and JG performed isolation compounds; SR, NA, and JM carried out biological assays. RA ointment preparation, while FE, WQ, and GE carried out the structural analysis. FE, SR, WQ, and FT wrote the manuscript.

## Funding

Authors thank Minciencias (Colombia) for the financial support through the grant 111571249866.

## Conflict of Interest

The authors declare that the research was conducted in the absence of any commercial or financial relationships that could be construed as a potential conflict of interest.
