# Supplementary material for: Exploring Antiparasitic Molecule Sources from Timber by-Product Industries—Leishmanicidal and Trypanocidal Compounds from Clathrotropis brunnea Amshoff
Source: Front Pharmacol. 2020 Dec 24;11:584668. doi: 10.3389/fphar.2020.584668 (PMC7788003; doi:10.3389/fphar.2020.584668)
Supplement: Supplementary file 1 [file datasheet1.pdf]

SQB2-2-1-3
proton

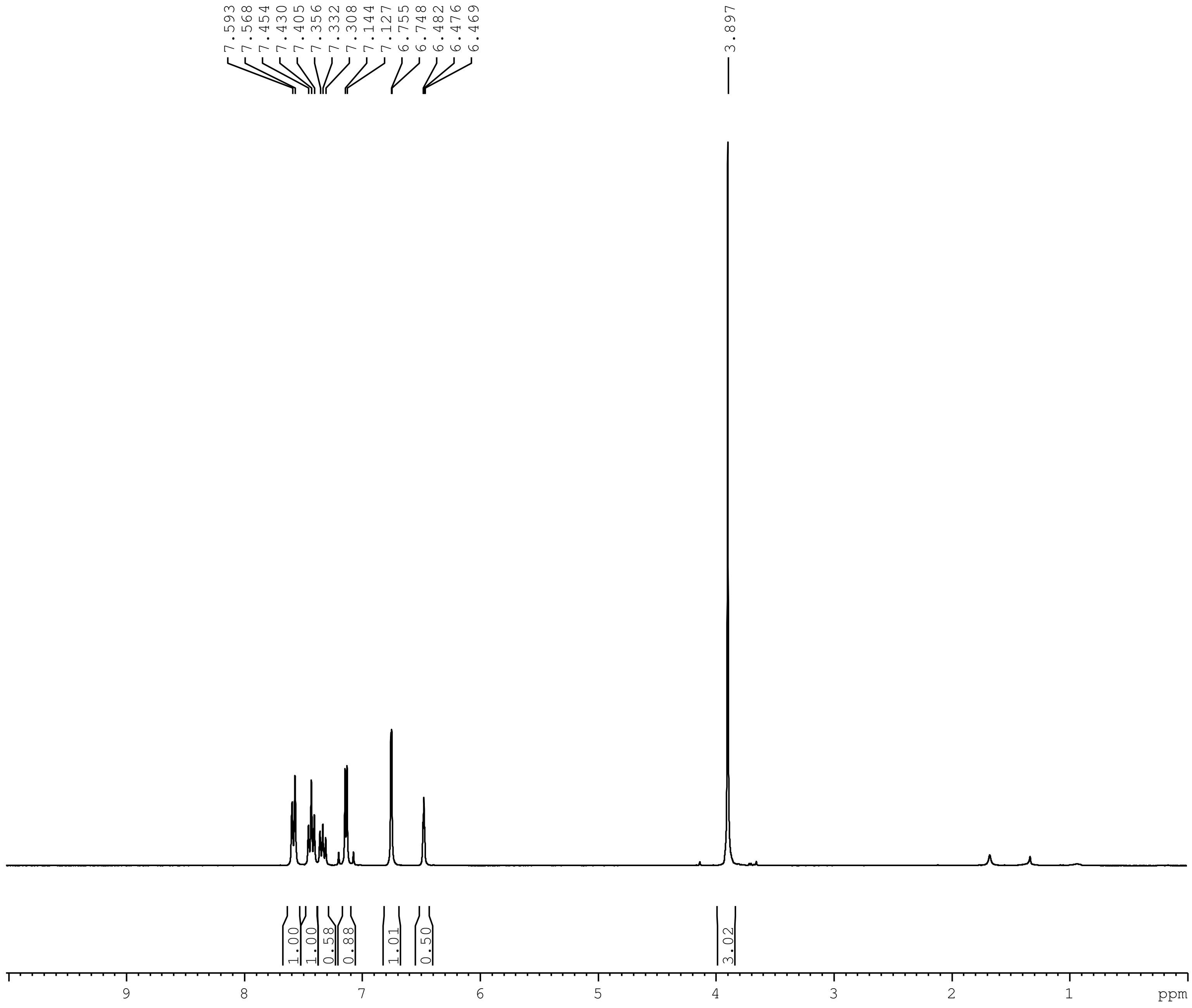

Current Data Parameters
NAME FT-SQB2-2-1-3
EXPNO 1
PROCNO 1

F2 - Acquisition Parameters
Date_ 20180308
Time 15.38
INSTRUM FOURIER300
PROBHD 5 mm DUL 13C-1
PULPROG zg30
TD 65536
SOLVENT CDCl3
NS 16
DS 0
SWH 6103.516 Hz
FIDRES 0.093132 Hz
AQ 5.3687091 sec
RG 14.3384
DW 81.920 usec
DE 6.50 usec
TE 293.7 K
D1 1.00000000 sec
TD0 1

======== CHANNEL f1 ========
SFO1 300.1698537 MHz
NUC1 1H
P1 9.16 usec
PLW1 23.00000000 W

F2 - Processing parameters
SI 65536
SF 300.1680000 MHz
WDW EM
SSB 0
LB 0.30 Hz
GB 0
PC 1.00

SQB2-2-1-3
Cosy

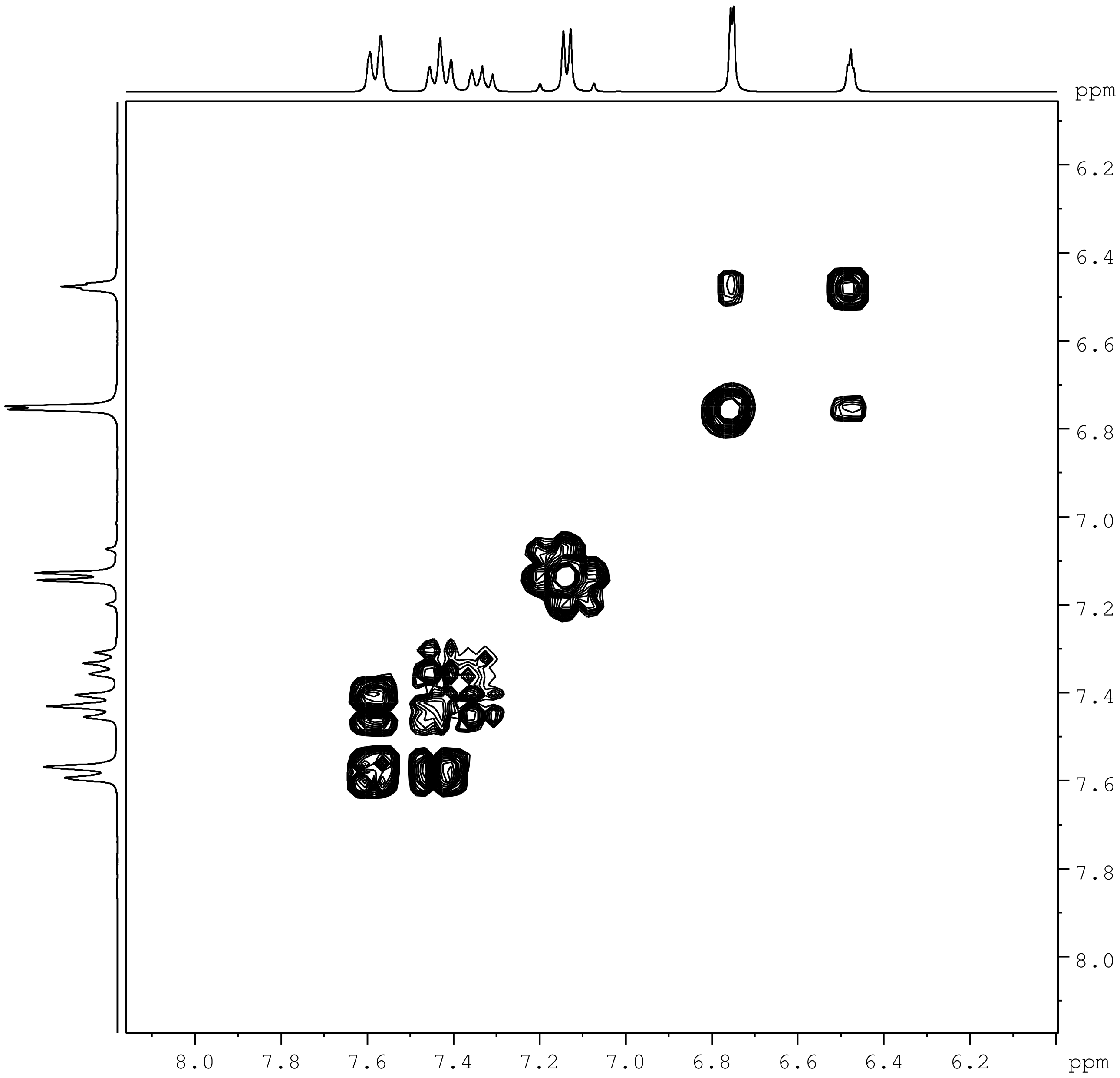

BRUKER

```
Current Data Parameters
NAME        FT-SQB2-2-1-3
EXPNO                   2
PROCNO                  1

F2 - Acquisition Parameters
Date_            20180308
Time                16.29
INSTRUM        FOURIER300
PROBHD     5 mm DUL 13C-1
PULPROG          cosygpqf
TD                   2048
SOLVENT             CDCl3
NS                      8
DS                      8
SWH              6103.516 Hz
FIDRES           2.980232 Hz
AQ              0.1677722 sec
RG                7.73804
DW                 81.920 usec
DE                   6.50 usec
TE                  293.4 K
D0             0.00000300 sec
D1             2.00000000 sec
D13            0.00000400 sec
D16            0.00020000 sec
IN0            0.00016380 sec

======== CHANNEL f1 ========
SFO1          300.1693808 MHz
NUC1                   1H
P0                   9.16 usec
P1                   9.16 usec
PLW1          23.00000000 W

====== GRADIENT CHANNEL =====
GPNAM[1]           RECT.1
GPZ1                10.00 %
P16               1000.00 usec

F1 - Acquisition parameters
TD                    256
SFO1             300.1694 MHz
FIDRES          23.847681 Hz
SW                 20.339 ppm
FnMODE                 QF

F2 - Processing parameters
SI                   1024
SF            300.1680000 MHz
WDW                 QSINE
SSB      0
LB       0 Hz
GB       0
PC                   1.40

F1 - Processing parameters
SI                   1024
MC2                    QF
SF            300.1680000 MHz
WDW                 QSINE
SSB      0
LB       0 Hz
GB       0
```

SQB2-2-1-3
Carbono 13

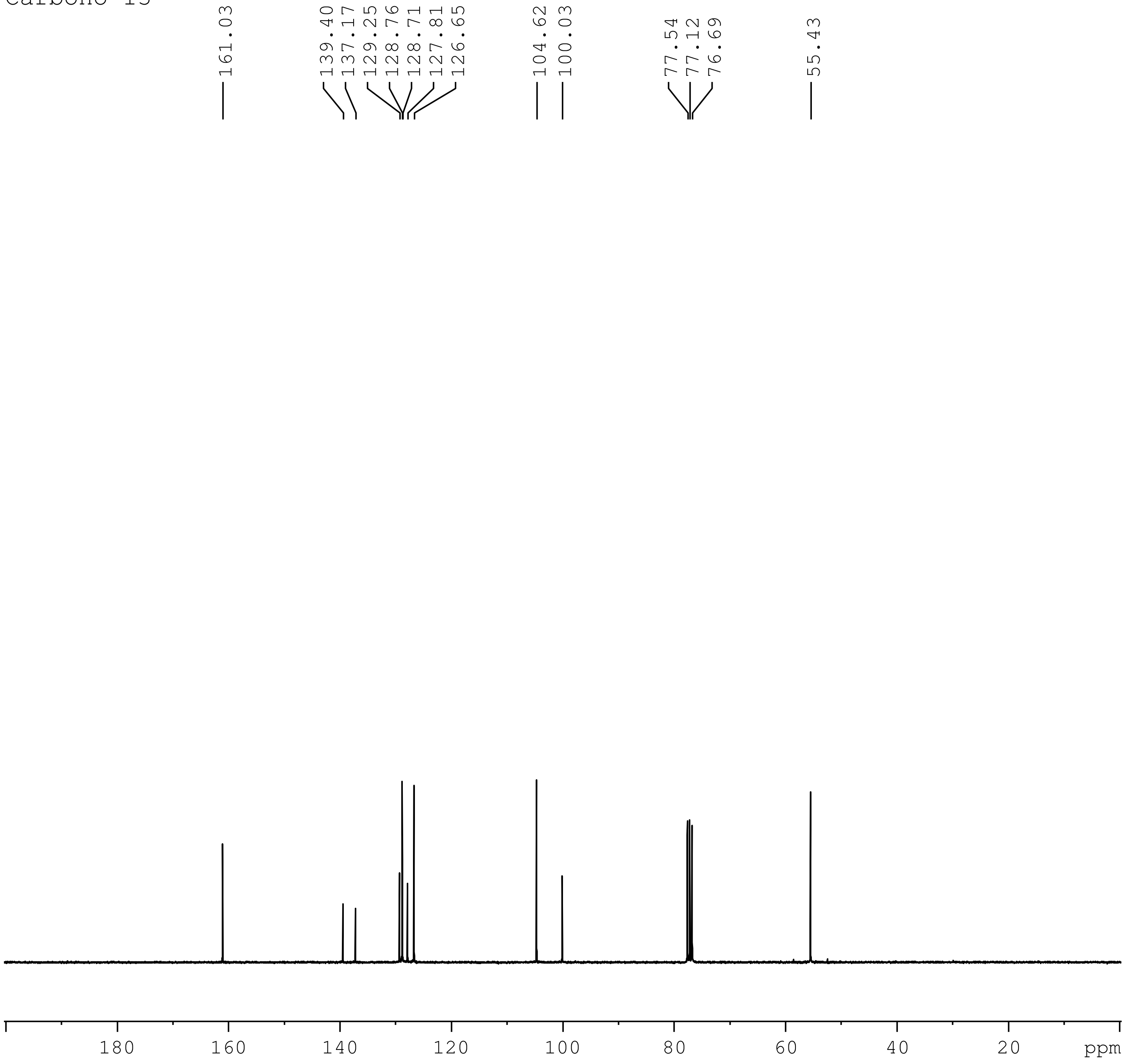

Current Data Parameters
NAME FT-SQB2-2-1-3
EXPNO 6
PROCNO 1

F2 - Acquisition Parameters
Date_ 20180309
Time 0.01
INSTRUM FOURIER300
PROBHD 5 mm DUL 13C-1
PULPROG zgpg30
TD 32768
SOLVENT CDCl3
NS 3072
DS 4
SWH 24414.063 Hz
FIDRES 0.745058 Hz
AQ 0.6710886 sec
RG 501.187
DW 20.480 usec
DE 6.50 usec
TE 293.8 K
D1 2.00000000 sec
D11 0.03000000 sec
D31 0.00001135 sec
D40 0.02898005 sec
L4 20
L5 57
P32 90.00 usec
TD0 1

======== CHANNEL f1 ========
SFO1 75.4848517 MHz
NUC1 13C
P1 11.35 usec
PLW1 25.00300026 W

======== CHANNEL f2 ========
SFO2 300.1692007 MHz
NUC2 1H
CPDPRG[2 waltz16
PCPD2 90.00 usec
PLW2 23.00000000 W
PLW12 0.31305999 W
PLW13 0.25356999 W

F2 - Processing parameters
SI 32768
SF 75.4773040 MHz
WDW EM
SSB 0
LB 1.00 Hz
GB 0
PC 1.40

SQB2-2-1-3
dept

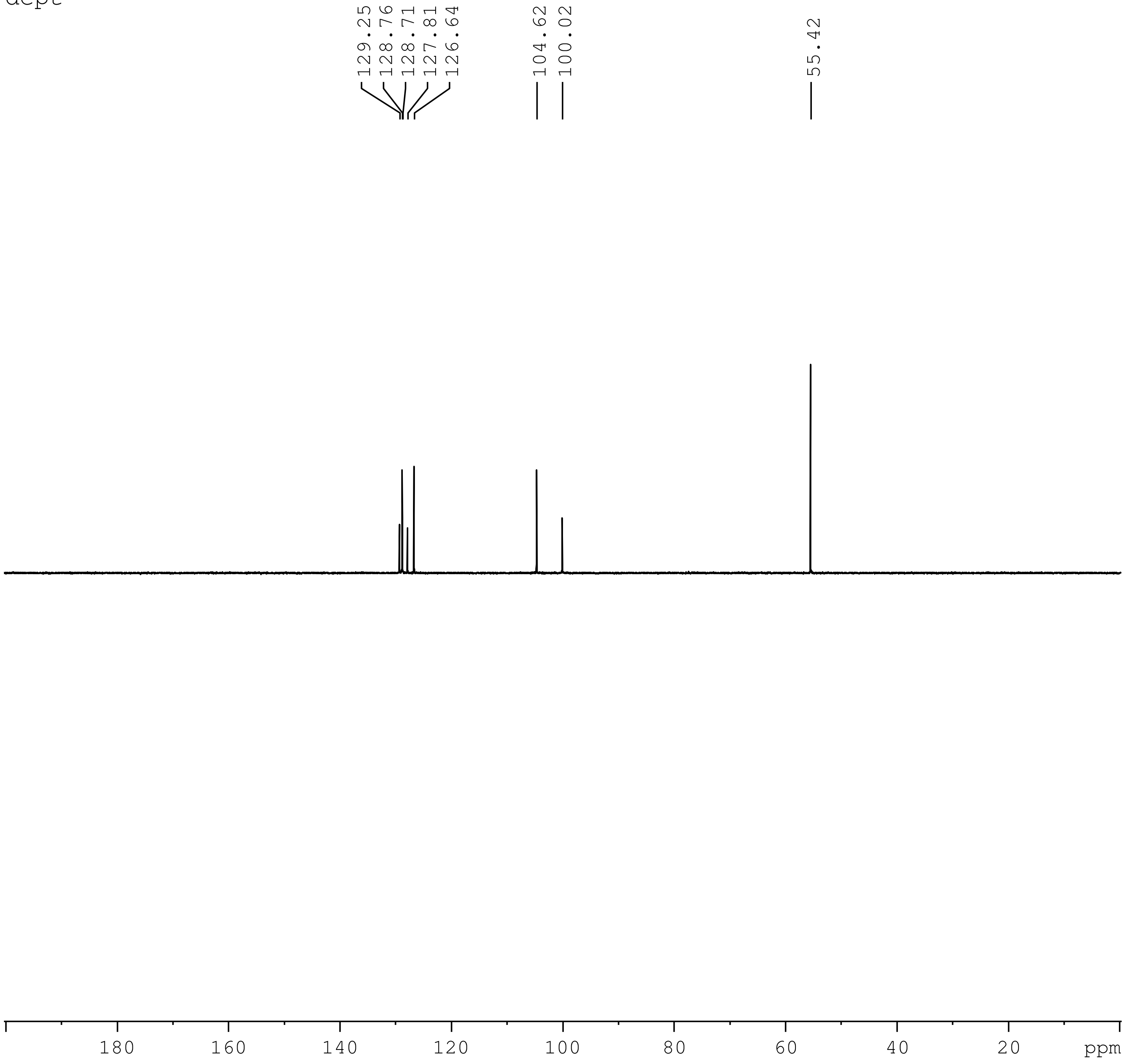


```
Current Data Parameters
NAME       FT-SQB2-2-1-3
EXPNO                  5
PROCNO                 1

F2 - Acquisition Parameters
Date_           20180308
Time               23.38
INSTRUM      FOURIER300
PROBHD   5 mm DUL 13C-1
PULPROG          dept135
TD                 32768
SOLVENT            CDCl3
NS                   512
DS                     4
SWH            24414.063 Hz
FIDRES          0.745058 Hz
AQ             0.6710886 sec
RG               501.187
DW                20.480 usec
DE                  6.50 usec
TE                 293.6 K
CNST2        145.0000000
D1            2.00000000 sec
D2            0.00344828 sec
D14           0.00001445 sec
D33           0.00001050 sec
D34           0.00343863 sec
D35           0.00345523 sec
L4                    20
P32                90.00 usec
TD0                    1

======== CHANNEL f1 ========
SFO1          75.4833422 MHz
NUC1                 13C
P1                 11.35 usec
P2                 22.70 usec
PLW1         25.00300026 W

======== CHANNEL f2 ========
SFO2         300.1692007 MHz
NUC2                  1H
CPDPRG[2         waltz16
P3                 10.50 usec
P4                 21.00 usec
PCPD2              90.00 usec
PLW2         23.00000000 W
PLW12         0.31305999 W

F2 - Processing parameters
SI                 32768
SF            75.4773040 MHz
WDW                   EM
SSB      0
LB                  1.00 Hz
GB       0
PC                  1.40
```

SQB2-2-1-3
hsqc

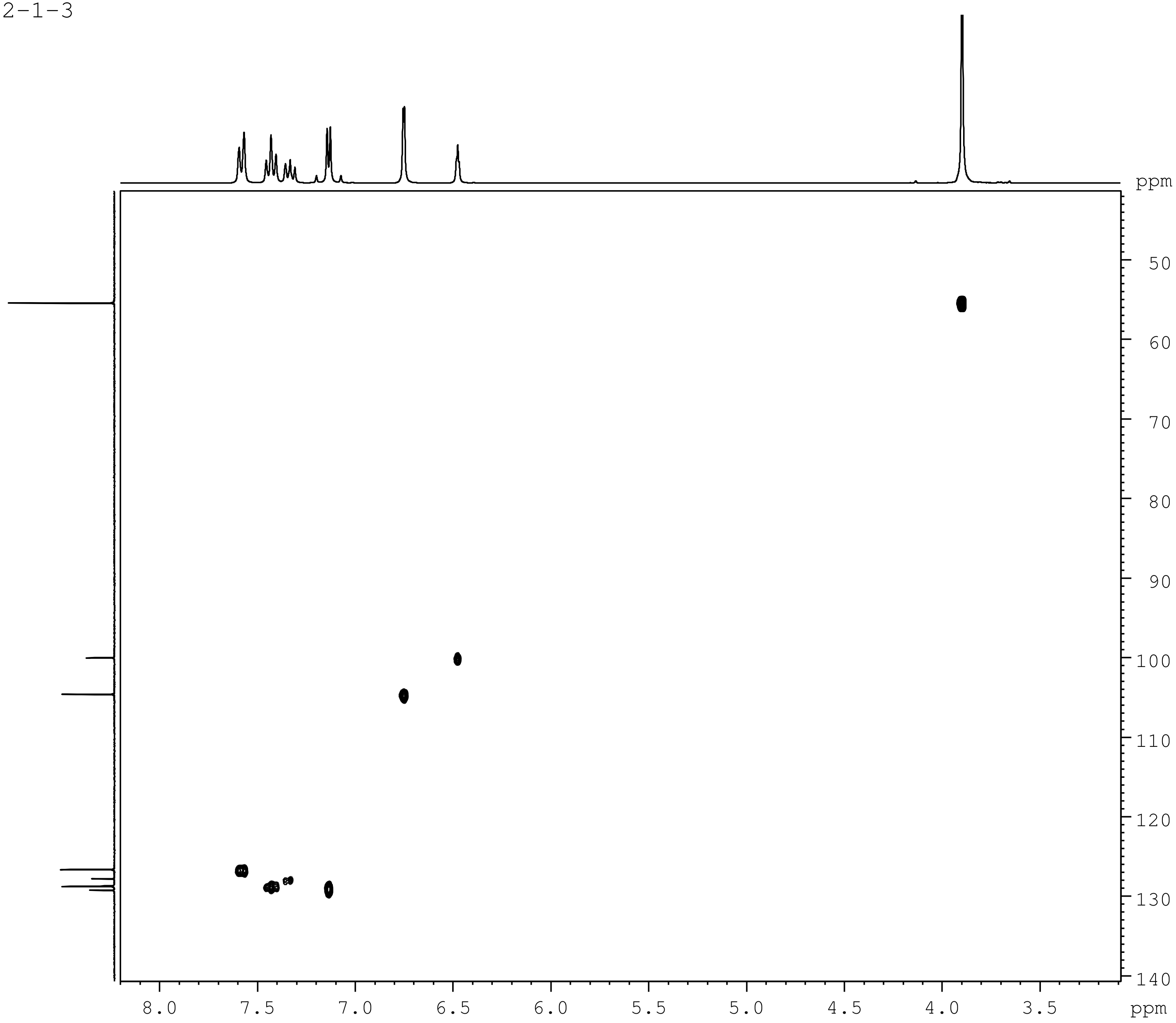

Current Data Parameters

NAME	FT-SQB2-2-1-3
EXPNO	3
PROCNO	1

F2 - Acquisition Parameters

Date_	20180308
Time	17.45
INSTRUM	FOURIER300
PROBHD	5 mm DUL 13C-1
PULPROG	hsqcgpph
TD	2048
SOLVENT	CDCl3
NS	16
DS	16
SWH	6103.516 Hz
FIDRES	2.980232 Hz
AQ	0.1677722 sec
RG	501.187
DW	81.920 usec
DE	6.50 usec
TE	293.4 K
CNST2	145.0000000
D0	0.00000300 sec
D1	1.50000000 sec
D4	0.00172414 sec
D11	0.03000000 sec
D13	0.00000400 sec
D16	0.00020000 sec
D31	0.00000916 sec
D32	0.00001832 sec
D33	0.00001135 sec
D34	0.00002270 sec
D36	0.00100000 sec
D37	0.00000110 sec
D38	0.00001325 sec
D42	0.00000219 sec
D43	0.00002432 sec
D44	0.00120000 sec
D45	0.00051614 sec
IN0	0.00002650 sec
L4	19
P35	74.00 usec

======== CHANNEL f1 ========

SFO1	300.1704013 MHz
NUC1	1H
P1	9.16 usec
P2	18.32 usec
PLW1	23.00000000 W

======== CHANNEL f2 ========

SFO2	75.4847763 MHz
NUC2	13C
CPDPRG[2	garp
P3	11.35 usec
P4	22.70 usec
PCPD2	74.00 usec
PLW2	25.00300026 W
PLW12	0.58819002 W

====== GRADIENT CHANNEL =====

GPNAM[1]	RECT.1
GPNAM[2]	RECT.1
GPNAM[3]	RECT.1
GPZ1	80.00 %
GPZ2	30.00 %
GPZ3	20.10 %
P16	1000.00 usec

F1 - Acquisition parameters

TD	256
SFO1	75.48478 MHz
FIDRES	73.702827 Hz
SW	249.957 ppm
FnMODE	TPPI

F2 - Processing parameters

SI	4096
SF	300.1680000 MHz
WDW	QSINE
SSB	2
LB	0 Hz
GB	0
PC	1.40

F1 - Processing parameters

SI	512
MC2	TPPI
SF	75.4773040 MHz
WDW	QSINE
SSB	2
LB	0 Hz
GB	0

SQB2-2-1-3
hmbc

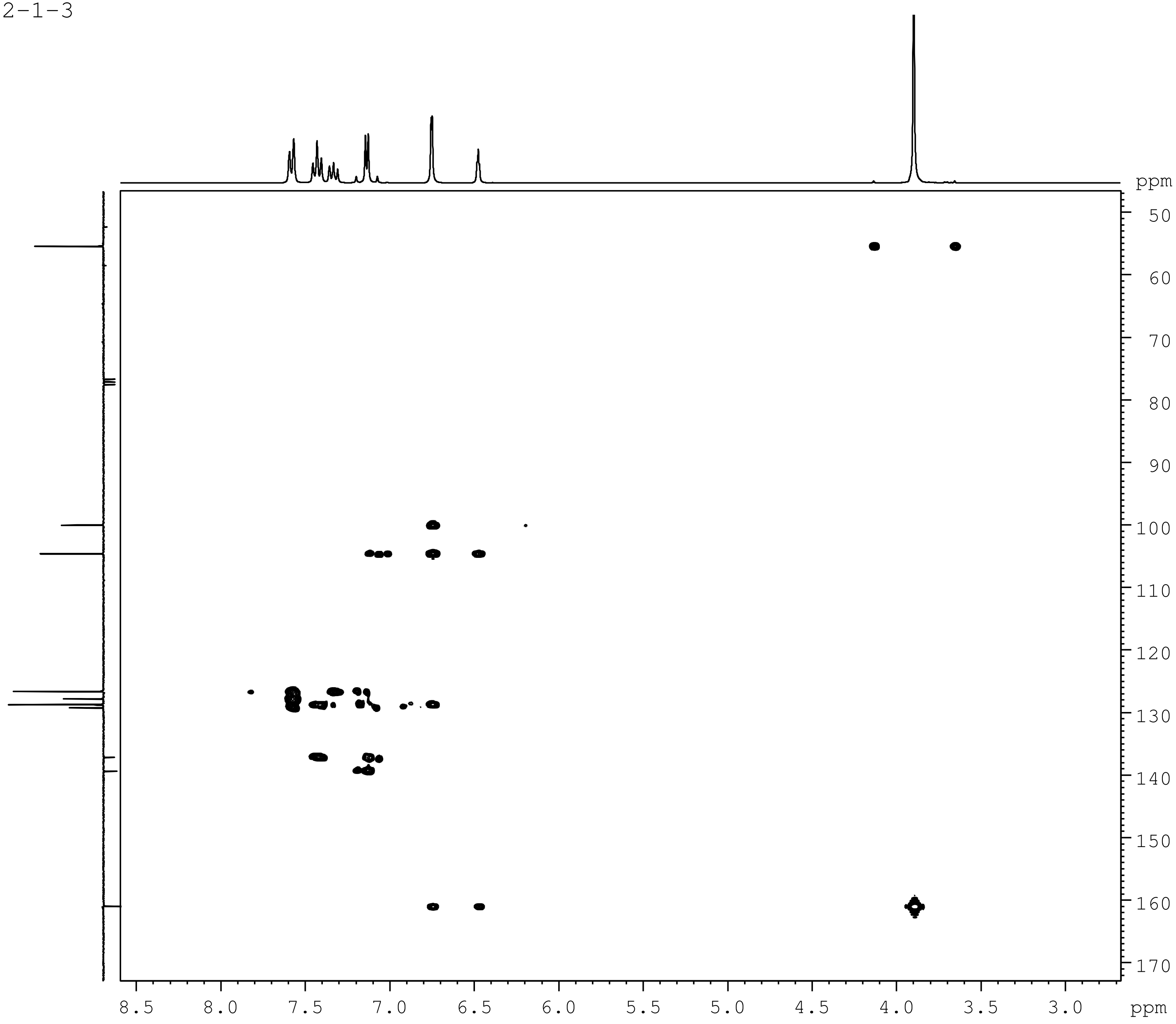

Current Data Parameters
NAME FT-SQB2-2-1-3
EXPNO 4
PROCNO 1

F2 - Acquisition Parameters
Date_ 20180308
Time 19.41
INSTRUM FOURIER300
PROBHD 5 mm DUL 13C-1
PULPROG hmbcgplpndqf
TD 2048
SOLVENT CDCl3
NS 32
DS 16
SWH 6103.516 Hz
FIDRES 2.980232 Hz
AQ 0.1677722 sec
RG 501.187
DW 81.920 usec
DE 6.50 usec
TE 294.0 K
CNST2 145.0000000
CNST13 10.0000000
D0 0.00000300 sec
D1 1.50000000 sec
D2 0.00344828 sec
D6 0.05000000 sec
D16 0.00020000 sec
D31 0.00000916 sec
D32 0.00001832 sec
D33 0.00001135 sec
D36 0.00100000 sec
D38 0.00002980 sec
IN0 0.00002980 sec

======== CHANNEL f1 ========
SFO1 300.1701012 MHz
NUC1 1H
P1 9.16 usec
P2 18.32 usec
PLW1 23.00000000 W

======== CHANNEL f2 ========
SFO2 75.4848517 MHz
NUC2 13C
P3 11.35 usec
PLW2 25.00300026 W

====== GRADIENT CHANNEL =====
GPNAM[1] RECT.1
GPNAM[2] RECT.1
GPNAM[3] RECT.1
GPZ1 50.00 %
GPZ2 30.00 %
GPZ3 40.10 %
P16 1000.00 usec

F1 - Acquisition parameters
TD 256
SFO1 75.48485 MHz
FIDRES 65.541107 Hz
SW 222.277 ppm
FnMODE QF

F2 - Processing parameters
SI 2048
SF 300.1680000 MHz
WDW SINE
SSB 0
LB 0 Hz
GB 0
PC 1.40

F1 - Processing parameters
SI 1024
MC2 QF
SF 75.4773040 MHz
WDW SINE
SSB 0
LB 0 Hz
GB 0
