# Supplementary material for: Exploring Antiparasitic Molecule Sources from Timber by-Product Industries—Leishmanicidal and Trypanocidal Compounds from Clathrotropis brunnea Amshoff
Source: Front Pharmacol. 2020 Dec 24;11:584668. doi: 10.3389/fphar.2020.584668 (PMC7788003; doi:10.3389/fphar.2020.584668)
Supplement: Supplementary file 2 [file datasheet2.pdf]

SQB2-2-1-4
proton

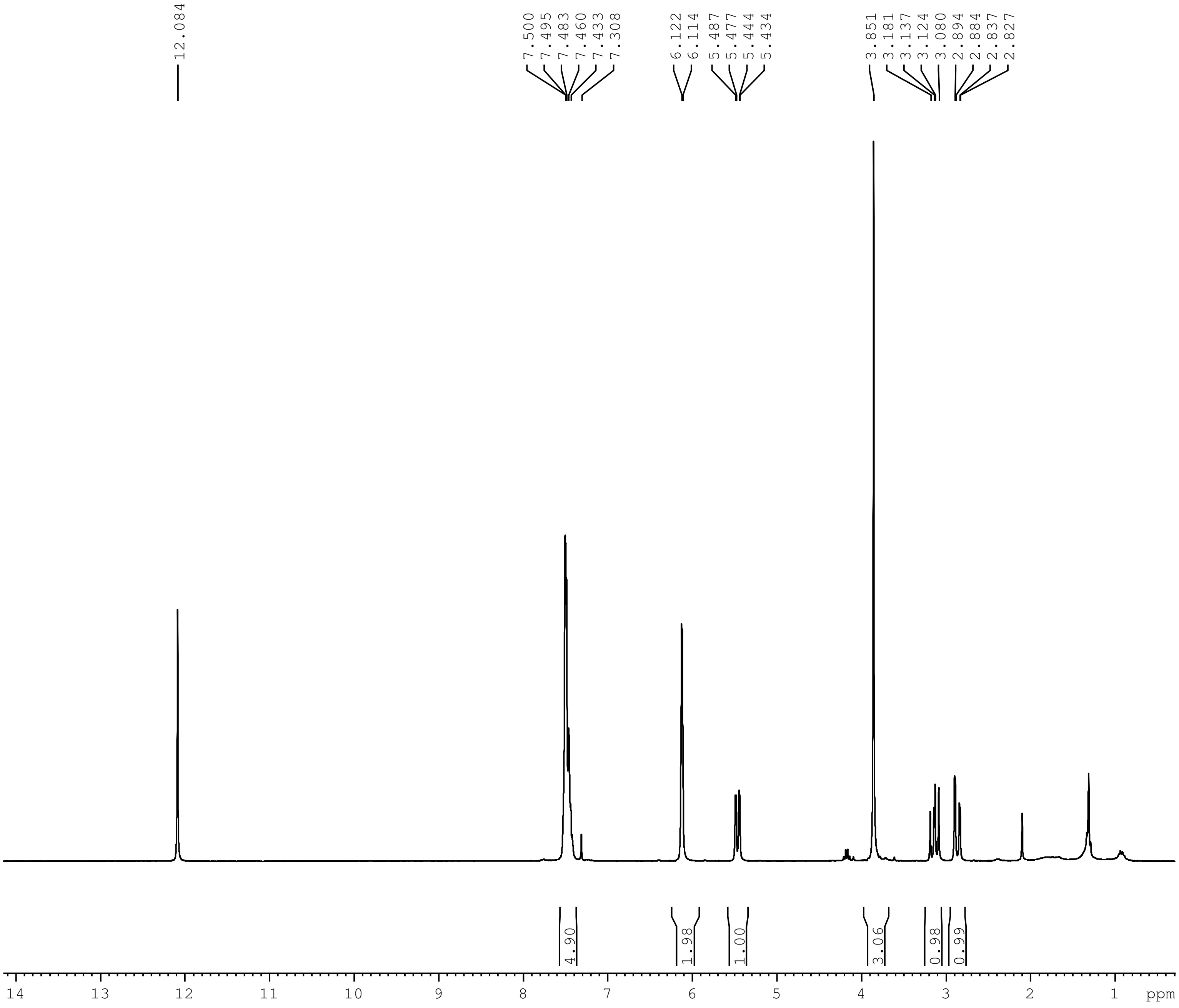

Current Data Parameters
NAME ACV-SQB-2-2(6)
EXPNO 111
PROCNO 1

F2 - Acquisition Parameters
Date_ 20180308
Time 15.57
INSTRUM FOURIER300
PROBHD 5 mm DUL 13C-1
PULPROG zg30
TD 65536
SOLVENT CDCl3
NS 16
DS 0
SWH 6103.516 Hz
FIDRES 0.093132 Hz
AQ 5.3687091 sec
RG 15.2457
DW 81.920 usec
DE 6.50 usec
TE 293.7 K
D1 1.00000000 sec
TD0 1

======== CHANNEL f1 ========
SFO1 300.1698537 MHz
NUC1 1H
P1 9.16 usec
PLW1 23.00000000 W

F2 - Processing parameters
SI 65536
SF 300.1680000 MHz
WDW EM
SSB 0
LB 0.30 Hz
GB 0
PC 1.00

SQB2-2-1-4
APT

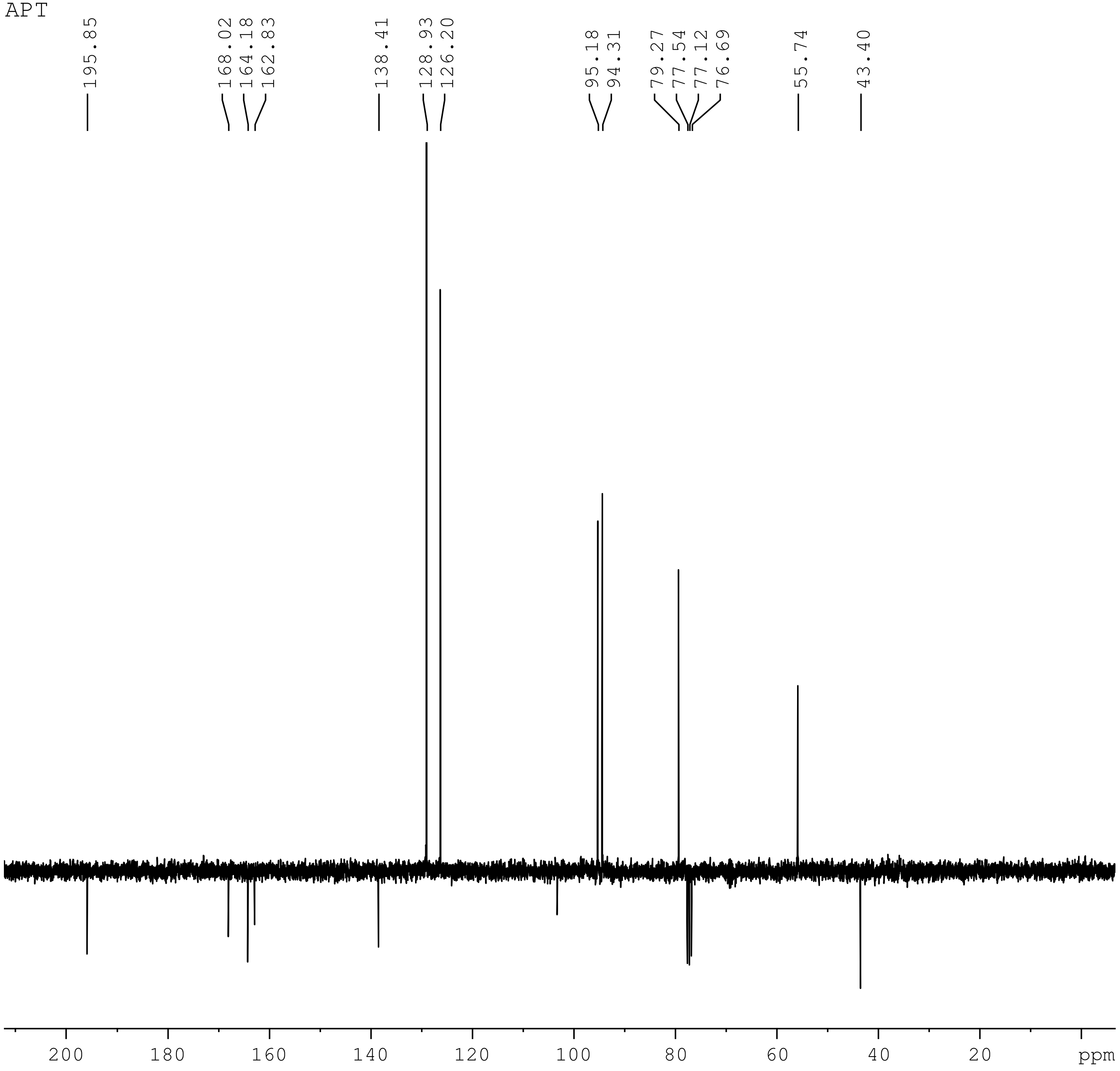

Current Data Parameters
NAME ACV-SQB-2-2(6)
EXPNO 17
PROCNO 1

F2 - Acquisition Parameters
Date_ 20180308
Time 15.59
INSTRUM FOURIER300
PROBHD 5 mm DUL 13C-1
PULPROG aptjc
TD 32768
SOLVENT CDCl3
NS 136
DS 4
SWH 24414.063 Hz
FIDRES 0.745058 Hz
AQ 0.6710886 sec
RG 501.187
DW 20.480 usec
DE 6.50 usec
TE 293.8 K
CNST2 145.0000000
D1 2.00000000 sec
D2 0.00344828 sec
D4 0.00172414 sec
D11 0.03000000 sec
D30 0.00001135 sec
D33 0.00001050 sec
D40 0.03008100 sec
D42 0.00002270 sec
D44 0.00002100 sec
L4 20
L5 57
P1 11.35 usec
P32 90.00 usec
TD0 1

======== CHANNEL f1 ========
SFO1 75.4848517 MHz
NUC1 13C
P0 11.35 usec
P2 22.70 usec
PLW1 25.00300026 W

======== CHANNEL f2 ========
SFO2 300.1692007 MHz
NUC2 1H
CPDPRG[2 waltz16
P3 10.50 usec
P4 21.00 usec
PCPD2 90.00 usec
PLW2 23.00000000 W
PLW12 0.31305999 W

F2 - Processing parameters
SI 32768
SF 75.4773040 MHz
WDW EM
SSB 0
LB 1.00 Hz
GB 0
PC 1.40

SQB2-2-1-4
13C DEPT-135

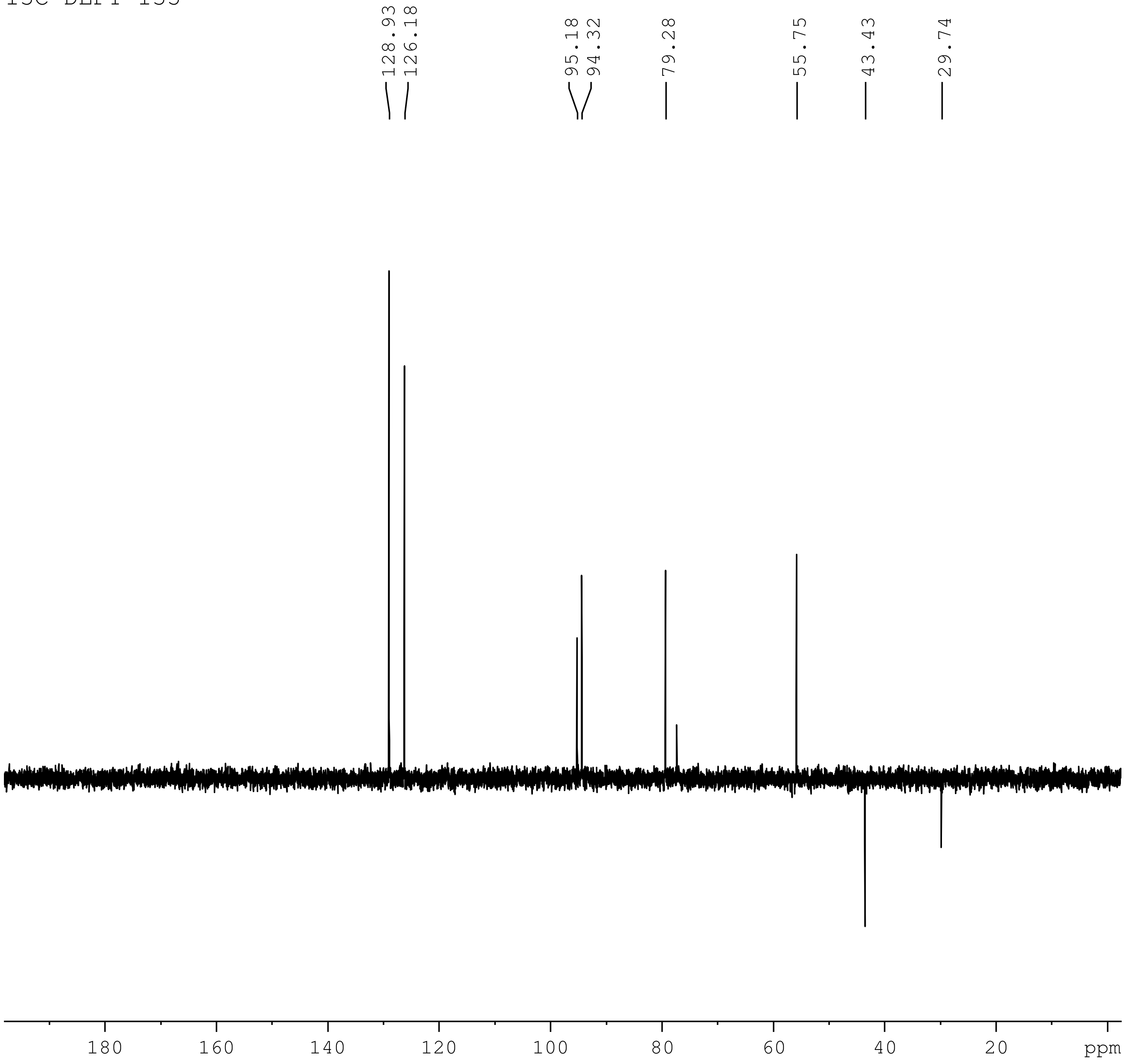

Current Data Parameters

NAME	ACV-SQB-2-2(6)
EXPNO	15
PROCNO	1

F2 - Acquisition Parameters

Date_	20180118
Time	21.52
INSTRUM	FOURIER300
PROBHD	5 mm DUL 13C-1
PULPROG	dept135
TD	65536
SOLVENT	CDCl3
NS	6144
DS	4
SWH	24414.063 Hz
FIDRES	0.372529 Hz
AQ	1.3421773 sec
RG	501.187
DW	20.480 usec
DE	6.50 usec
TE	293.8 K
CNST2	145.0000000
D1	2.00000000 sec
D2	0.00344828 sec
D14	0.00001592 sec
D33	0.00001275 sec
D34	0.00343528 sec
D35	0.00345415 sec
L4	40
P32	90.00 usec
TD0	1

======== CHANNEL f1 ========

SFO1	75.4833422 MHz
NUC1	13C
P1	12.50 usec
P2	25.00 usec
PLW1	25.00300026 W

======== CHANNEL f2 ========

SFO2	300.1692007 MHz
NUC2	1H
CPDPRG[2	waltz16
P3	12.75 usec
P4	25.50 usec
PCPD2	90.00 usec
PLW2	12.00000000 W
PLW12	0.24083000 W

F2 - Processing parameters

SI	32768
SF	75.4773040 MHz
WDW	EM
SSB	0
LB	1.00 Hz
GB	0
PC	1.40

SQB2-2-1-4
13C-1H HMBC

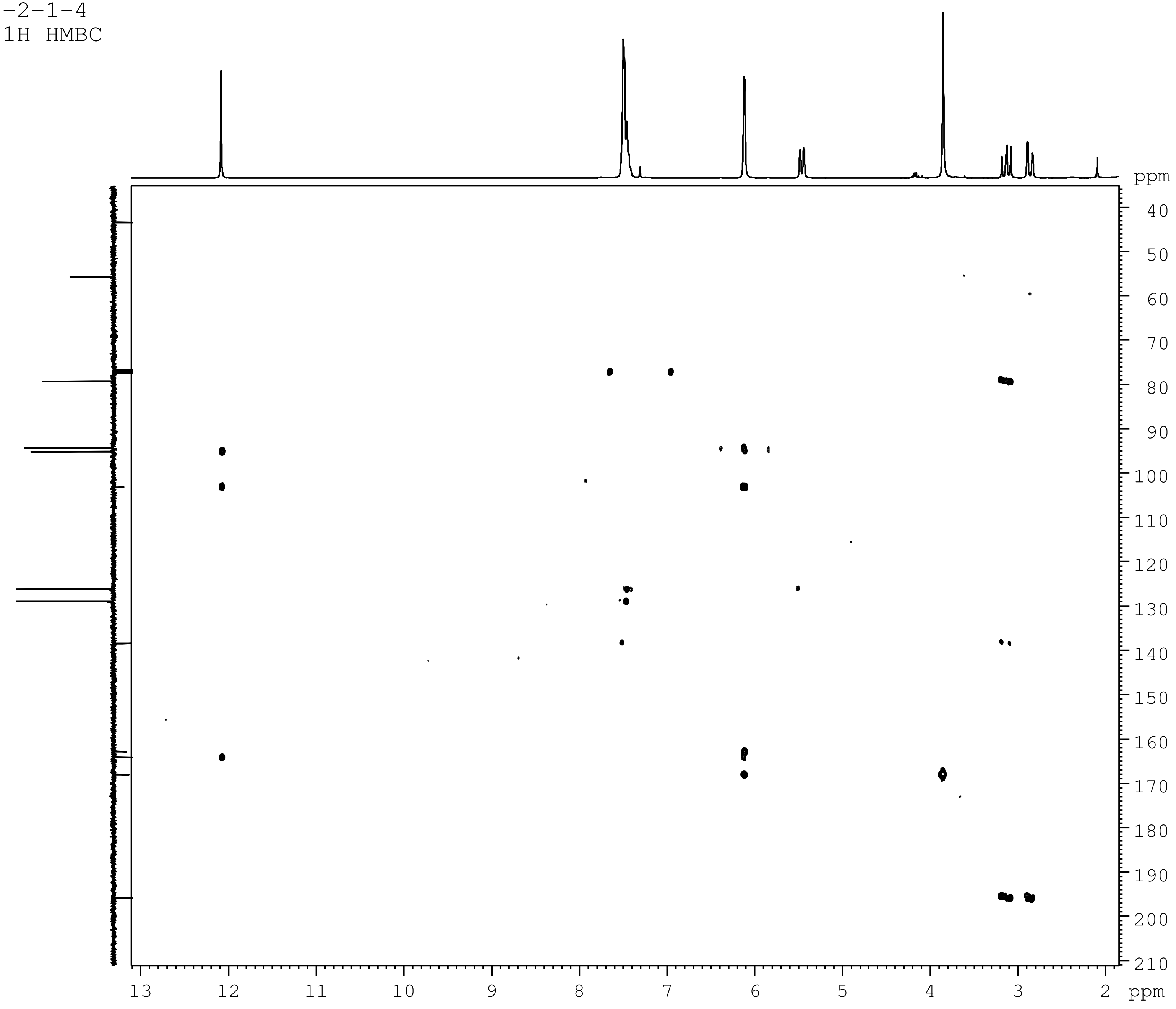


```
Current Data Parameters
NAME      ACV-SQB-2-2(6)
EXPNO                 12
PROCNO                 1

F2 - Acquisition Parameters
Date_           20180118
Time               17.54
INSTRUM       FOURIER300
PROBHD   5 mm DUL 13C-1
PULPROG    hmbcgplpndqf
TD                  2048
SOLVENT            CDCl3
NS                    32
DS                    16
SWH             6103.516 Hz
FIDRES          2.980232 Hz
AQ             0.1677722 sec
RG               501.187
DW                81.920 usec
DE                  6.50 usec
TE                 294.2 K
CNST2        145.0000000
CNST13        10.0000000
D0            0.00000300 sec
D1            1.50000000 sec
D2            0.00344828 sec
D6            0.05000000 sec
D16           0.00020000 sec
D31           0.00000916 sec
D32           0.00001832 sec
D33           0.00001250 sec
D36           0.00100000 sec
D38           0.00002980 sec
IN0           0.00002980 sec

======== CHANNEL f1 ========
SFO1         300.1701012 MHz
NUC1                  1H
P1                  9.16 usec
P2                 18.32 usec
PLW1         30.00000000 W

======== CHANNEL f2 ========
SFO2          75.4848517 MHz
NUC2                 13C
P3                 12.50 usec
PLW2         25.00300026 W

====== GRADIENT CHANNEL =====
GPNAM[1]          RECT.1
GPNAM[2]          RECT.1
GPNAM[3]          RECT.1
GPZ1               50.00 %
GPZ2               30.00 %
GPZ3               40.10 %
P16              1000.00 usec

F1 - Acquisition parameters
TD                   256
SFO1            75.48485 MHz
FIDRES         65.541107 Hz
SW               222.277 ppm
FnMODE                QF

F2 - Processing parameters
SI                  2048
SF           300.1680000 MHz
WDW                 SINE
SSB       0
LB        0 Hz
GB        0
PC                  1.40

F1 - Processing parameters
SI                  1024
MC2                   QF
SF            75.4773040 MHz
WDW                 SINE
SSB       0
LB        0 Hz
GB        0
```
